# Clinicopathological features and prognosis of colonic gastrointestinal stromal tumors: evaluation of a pooled case series

**DOI:** 10.18632/oncotarget.9196

**Published:** 2016-05-05

**Authors:** Fan Feng, Yangzi Tian, Zhen Liu, Guanghui Xu, Shushang Liu, Man Guo, Xiao Lian, Daiming Fan, Hongwei Zhang

**Affiliations:** ^1^ Department of Digestive Surgery, Xijing Hospital, Fourth Military Medical University, Xi'an, Shaanxi, China; ^2^ Department of Dermatology, Xijing Hospital, Fourth Military Medical University, Xi'an, Shaanxi, China

**Keywords:** gastrointestinal stromal tumor, colon, feature, prognosis

## Abstract

**Background:**

Due to the extremely rare incidence, data about colonic GISTs are limited. Therefore, aim of the present study was to explore clinicopathological characteristics and prognosis of colonic GISTs.

**Patients and Methods:**

Colonic GISTs cases were obtained from our center and from case report and clinical studies extracted from MEDLINE. Clinicopathological features and survivals were analyzed.

**Results:**

There were 79 colonic GISTs patients with a female predominance. The median age was 66 years (range 0.17-84). The median tumor size was 5.8 cm (range 0.5-29). The most common location was sigmoid colon (45.8%), followed by transverse colon (19.5%). The majority of colonic GISTs were high risk (70.8%). Mitotic index was correlated with gender (*P* = 0.002) and tumor size (*P* = 0.005), and tumor location was correlated with age (*P* = 0.017). The five year DFS and DSS were 57.4% and 61.6%, respectively. Mitotic index and NIH risk classification were associated with prognosis of colonic GISTs. However, mitotic index was the only independent risk factor. The distribution of tumor size and NIH risk classification were significantly different between colonic and gastric GISTs (both *P* = 0.000). The DFS and DSS of colonic GISTs were significantly lower than that of gastric GISTs (*P* = 0.012 and *P* = 0.002, respectively).

**Conclusions:**

The most common location for colonic GISTs was sigmoid colon. Most tumors were high risk. Mitotic index was the only independent risk factor for prognosis of colonic GISTs. Colonic GISTs differ significantly from gastric GISTs in respect to clinicopathological features. The prognosis of colonic GISTs was worse than that of gastric GISTs.

## INTRODUCTION

Gastrointestinal stromal tumors (GISTs) are commonest mesenchymal neoplasm of alimentary tract, represent 1% to 2% of alimentary malignant tumors [1]. GISTs are derived from interstitial cells of Cajal (ICC), and are related with activating mutations in KIT protooncogene [2]. It has been established through positive staining for CD117 and CD34 [3]. Histologically, most GISTs show spindle morphology (70%), followed by epithelioid (20%) and mixed morphology (10%) [4].

GISTs can occur anywhere in the alimentary tract but most commonly in the stomach (40% to 70%) [5]. GISTs located in the colorectum are relatively rare, representing approximately 5% of all GISTs [6]. GISTs located in colon is much rarer, and it represents only 1-2% of all cases [7]. Thus, studies involving large numbers of colonic GISTs are lacking, and the clinicopathological profiles and prognosis are limited. Therefore, the aim of our present study was to explore clinicopathological characteristics and prognosis of colonic GISTs.

## RESULTS

The clinicopathological characteristics were summarized in Table 1. There were 34 male (43%) and 41 female (57%). The patient age ranged from 0.17-84 years (mean, 60.9 years; median, 66 years). Eight patients accompanied with GISTs in other locations (12.5%), including 2 cases with liver metastasis, 3 cases with peritoneal metastasis, 2 cases with rectal GIST and one case with jejunal GIST. Three patients accompanied with other malignant tumors (4.7%), including 2 cases of ascending colon cancer and one case of endometrial carcinoma. The most common symptom was abdominal pain (16/47, 34.0%), followed by obstruction (11/47, 23.4%), bleeding (11/47, 23.4%), perforation (7/47, 13.9%) and abdominal mass (6/47, 12.8%). The most common location was sigmoid colon (33/72, 45.8%), followed by transverse colon (14/72, 19.5%), descending colon (9/72, 12.5%), ascending colon (8/72, 11.1%) and cecum (8/72, 11.1%). Sixty-seven patients underwent complete surgical resection (67/72, 93.1%), four patients underwent palliative surgical resection (4/72, 5.5%), and one patient treated with adjuvant imatinib therapy only (1/72, 1.4%).

**Table 1:** Clinicopathological characteristics of 79 cases of colonic GISTs

Characteristics	Number	Percentage
Age(∑=75)		
≤60	33	44.0%
>60	42	56.0%
Gender(∑=75)		
Male	34	43.0%
Female	41	57.0%
Accompanied tumor(∑=64)		
GISTs with other locations	8	12.5%
Other manignant tumors	3	4.7%
Symptoms(∑=47)		
Abdominal pain	16	34.0%
Obstruction	11	23.4%
Bleeding	11	23.4%
Perforation	7	13.9%
Abdominal mass	6	12.8%
Location(∑=72)		
Cecum	8	11.1%
Ascending colon	8	11.1%
Transverse colon	14	19.5%
Descending colon	9	12.5%
Sigmoid colon	33	45.8%
Surgical resection(∑=72)		
Complete resection	67	93.1%
Incomplete resection	4	5.5%
No surgery	1	1.4%
Tumor size(∑=69)		
≤2cm	14	20.3%
2.1-5cm	17	24.6%
5.1-10cm	23	33.3%
>10cm	15	21.8%
Mitotic index(∑=66)		
≤5	32	48.5%
>5	34	51.5%
Histological type(∑=56)		
Spindle	49	87.5%
Epithelioid	3	5.4%
Mixed	4	7.1%
Lymph node metastasis(∑=17)		
Yes	3	17.6%
No	14	82.4%
Immunohistochemisty		
CD117(∑=58)	48	82.8%
CD34(∑=44)	30	68.2%
DOG-1(∑=6)	4	66.7%
Mutational status(∑=23)		
KIT exon 11	10	43.5%
Others	13	56.5%
NIH risk category(∑=65)		
Very low risk	12	18.4%
Low risk	7	10.8%
Intermediate risk	0	0%
High risk	46	70.8%
Adjuvant therapy(∑=30)		
Yes	8	26.7%
No	22	73.3%

Tumor size ranged from 0.5 to 29 cm in maximum diameter (mean, 6.5 cm; median, 5.8 cm). The mitotic index of 34 patients exceeded 5/50 HPF (34/66, 51.5%). Forty-nine patients displayed spindle cell morphology (49/56, 87.5%), three patients displayed epithelioid morphology (3/56, 5.4%) and four patients displayed mixed morphology (4/56, 7.1%). Among the 17 patients with lymph node dissection, 3 patients had lymph node metastasis (3/17, 17.6%). CD117 positivity was detected in 48 patients (48/58, 82.8%), CD34 positivity was detected in 30 patients (30/44, 68.2%) and DOG-1 positivity was detected in 4 patients (4/6, 66.7%). Twenty-three patients were analyzed for gene mutation status. Ten patients carrying a mutation in exon 11 of KIT (10/23, 43.5%). According to NIH risk classification, 12 patients were very low risk (12/65, 18.4%), 7 patients were low risk (7/65, 10.8%), no patient was intermediate risk, and 46 patients were high risk (46/65, 70.8%). Adjuvant imatinib therapy were recorded in 30 patients, and 8 patients (26.7%) received imatinib therapy. Among them, one patient received imatinib therapy after biopsy, the remaining 7 patients received imatinib therapy after surgical resection.

**Figure 1:**
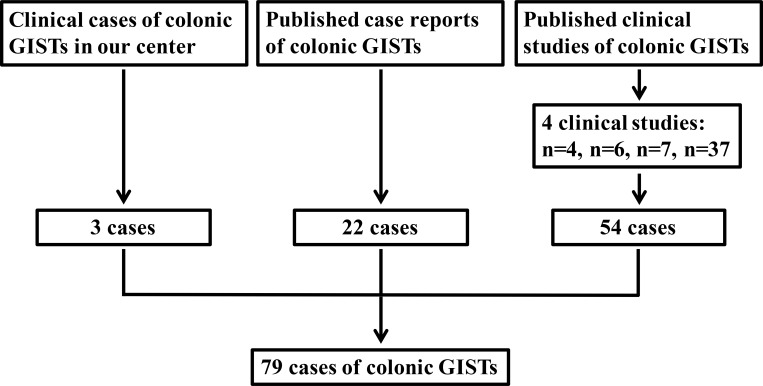
Schematic diagram regarding selection of colonic GISTs

**Figure 2:**
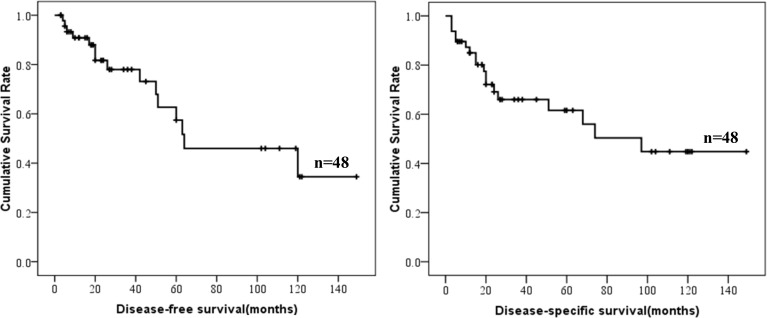
DFS and DSS of colonic GISTs

The relationship between clinicopathological features were summarized in Table 2. The mitotic index exceeded 5/50HPF for the majority of male patients but only for the minority of female patients (*P* = 0.002), and it was positively related with tumor size (*P* = 0.005). Age was associated with tumor location (*P* = 0.017). For patients less than 60 years old, the commonest location were transverse and sigmoid colon. For patients more than 60 years old, the commonest location was sigmoid colon only.

**Table 2:** The relationship between clinicopathological characteristics

Characteristics	Mitotic index(≤5)	Mitotic index(>5)	*P* value
Gender			
Male	7(23.3%)	20(62.5%)	0.002
Female	23(76.7%)	12(37.5%)	
Tumor size (cm)			
≤2	10(32.3%)	0(0%)	0.005
2.1-5	7(22.5%)	7(23.3%)	
5.1-10	10(32.3%)	13(43.4%)	
>10	4(12.9%)	10(33.3%)	
			
Tumor location	Age ≤60	Age >60	
Cecum	3(10.0%)	5(13.1%)	0.017
Ascending	3(10.0%)	3(7.9%)	
Transverse	11(36.7%)	2(5.3%)	
Descending	3(10.0%)	6(15.8%)	
Sigmoid	10(33.3%)	22(57.9%)	

Survival data of colonic GISTs were summarized in Table 3. Survival data of 48 patients were eventually selected for analysis. The median follow-up time was 23.5 months (range from 3 to 149 months). Fifteen cases showed recurrence or metastasis, 18 patients suffered from GIST related deaths. The 1-, 3- and 5-year DSS was 85.0%, 66.0% and 61.6%, respectively. The 1-, 3- and 5-year DFS was 90.8%, 78.0% and 57.4%, respectively. The DFS and DSS of colonic GISTs were shown in Figure 2.

**Table 3:** Survival data of 48 cases of colonic GISTs

Survival characteristics	Parameter
Follow up time	
Mean(m, ±SD)	41.69±40.63
Median(m, range)	23.5 (3-149)
Survival data	
Recurrence or metastasis	15
GISTs related deaths	18
Survival rates (%)	
1-/3-/5-year DSS	85.0/66.0/61.6
1-/3-/5-year DFS	90.8/78.0/57.4

**Figure 3:**
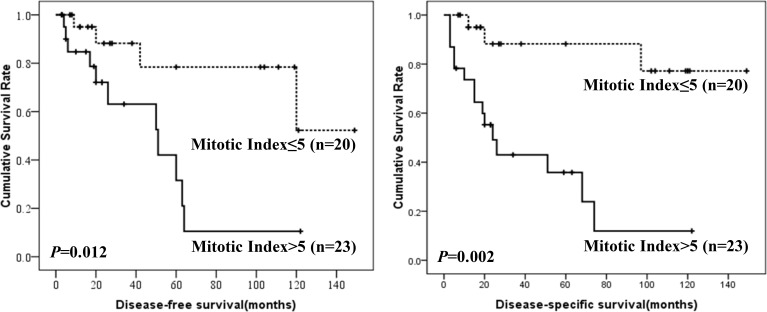
DFS and DSS of colonic GISTs by mitotic index

**Figure 4:**
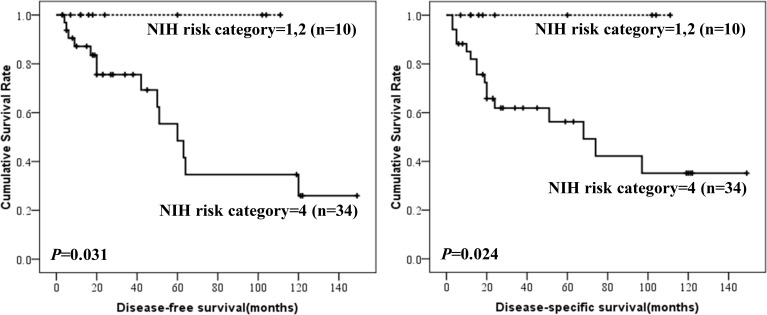
DFS and DSS of colonic GISTs by NIH risk category

Prognostic factors for DFS and DSS were shown in Table 4. Mitotic index and NIH risk classification were associated with prognosis of colonic GISTs. However, only mitotic index was independent risk factor. The DFS and DSS according to mitotic index and NIH risk classification were shown in Figures 3 and 4. NIH risk classification was not enrolled in logistic regression analysis, although it showed significant correlation with prognosis. Because no patients suffered from recurrence, metastasis or death in NIH risk category 1 and 2. When calculating log of odds ratio, the null frequency resulted in a computational error due to presence of logarithm of zero.

**Table 4:** Prognostic factors for DS and DFS in patients with colonic GISTs according to univariate and multivariate analysis

Prognostic factors	Univariate analysis			Multivariate analysis		
	β	Hazard ratio (95% CI)	*P* value	β	Hazard ratio (95% CI)	*P* value
DSS (*n* = 48)						
Tumor size(≤5/>5)	1.34	3.83 (0.87-16.87)	0.076			
Mitotic index(≤5/>5)	2.06	7.86 (2.17-28.47)	0.002	2.01	7.46 (1.88-29.63)	0.004
NIH risk category(1,2/4)	3.44	31.19 (0.23-4308.01)	0.024			
DFS (*n* = 48)						
Tumor size(≤5/>5)	0.43	1.54 (0.48-4.98)	0.468			
Mitotic index(≤5/>5)	1.48	4.40 (1.38-14.00)	0.012	1.43	4.17 (1.27-13.72)	0.019
NIH risk category(1,2/4)	3.50	32.96 (0.17-6477.05)	0.031			

**Figure 5:**
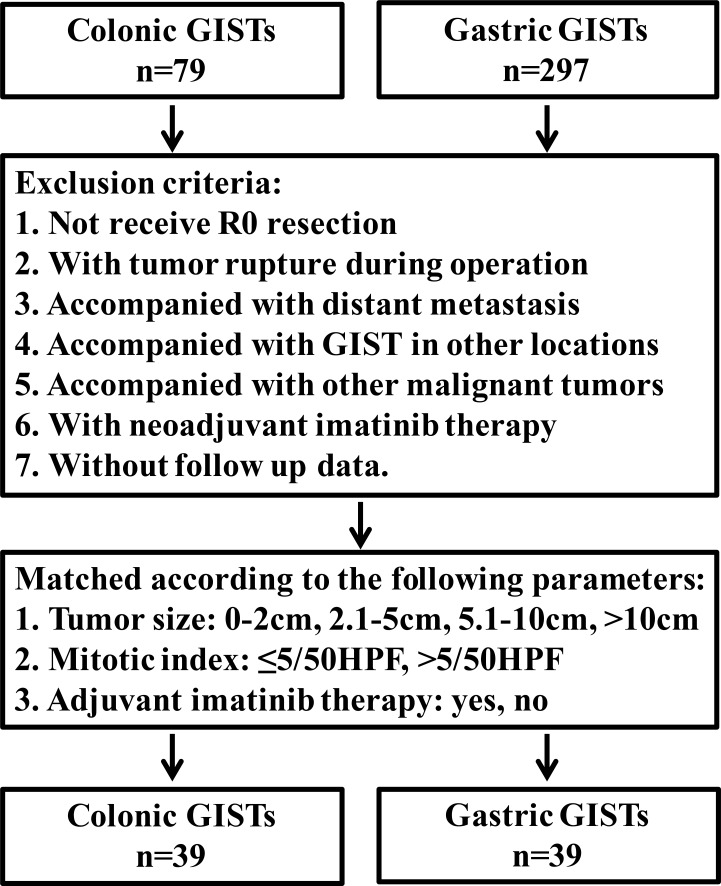
Flow chart of match strategy between colonic and gastric GISTs

The clinicophathological features of 79 colonic GISTs were compared with 297 gastric GISTs in our center (Table 5). The results showed that the distribution of tumor size and NIH risk classification were significantly different between colonic and gastric GISTs (both *P* = 0.000). In order to compare the prognosis between colonic and gastric GISTs, the two groups were matched according to tumor size, mitotic index and imatinib treatment. The entire process was shown in Figure 5. Finally, 39 cases of colonic GISTs and 39 cases of gastric GISTs were selected. No intergroup difference was found in age, gender, tumor size, mitotic index and imatinib treatment (Table 6). The DFS (*P* = 0.012) and DSS (*P* = 0.002) of colonic GISTs were significantly lower than that of gastric GISTs (56.0% *vs* 85.7%, 62.7% *vs* 96.3%).

**Table 5:** Comparison of selected clinicopathological parameters between colonic and gastric GISTs

Characteristics	Colon (*n* = 79)	Stomach(*n* = 297)	*P* value
Age			
≤60	33	168	0.053
>60	42	129	
Gender			
Male	34	155	0.304
Female	41	142	
Tumor size			
≤2cm	14	96	0.000
2.1-5cm	17	107	
5.1-10cm	23	72	
>10cm	15	22	
Histological type			
Spindle	49	275	0.067
Epithelioid	3	3	
Mixed	4	19	
Mitotic index			
≤5	32	163	0.346
>5	34	134	
NIH risk category			
Very low risk	12	83	0.000
Low risk	7	58	
Intermediate risk	0	87	
High risk	46	69	

**Table 6:** Comparison of predefined variables between colonic and gastric GISTs

Characteristics	Colon (*n*= 39)	Stomach(*n*= 39)	*P* value
Age			
≤60	17	18	1.000
>60	22	21	
Gender			
Male	15	16	1.000
Female	24	23	
Tumor size			
≤2cm	5	5	1.000
2.1-5cm	9	9	
5.1-10cm	17	17	
>10cm	8	8	
Mitotic index			
≤5	21	21	1.000
>5	18	18	
Adjuvant therapy			
Yes	2	2	1.000
No	37	37	

## DISCUSSION

GISTs located in the colon constitute a very rare subset with limited data on the clinicopathological features and prognosis. Therefore, we evaluated 79 cases of colonic GISTs from our center and from literatures in MEDLINE. The present study represents the largest analysis of colonic GISTs.

The study containing 37 cases of colonic GISTs reported by Miettinen et al. [8] was the only one with a much larger number of patients. In the series, the commonest site was sigmoid colon, followed by transverse colon, cecum, descending and ascending colon. In our present study, the commonest location was also sigmoid colon, and followed by transverse colon. However, the ratio of cecum, ascending and descending colon were almost equivalent. The distribution of GISTs may be attributed to the distribution of ICCs in the colon. However, Hagger et al. reported that the highest density of ICC was at the myenteric plexus of the transverse colon, not the sigmoid colon [9]. Further, we found that the distribution of colonic GISTs was correlated with age of patients. For patients less than 60 years old, the commonest location were transverse and sigmoid colon. For patients more than 60 years old, the commonest location was sigmoid colon only. This may be attributed to the decline of ICC number in the colon with age at a rate of 13% per decade reported by Gomez-Pinilla et al. [10]. They also found that volume of ICC networks decreased more quickly with age in the ascending colon than that in the sigmoid colon. Although the distribution of colonic GISTs in our present study could not fully elucidated by the above studies, they provide clues for the investigation of distribution of colonic GISTs.

The surgical treatment of GISTs is radical resection of the primary tumor with negative microscopic margins. It is well known that lymph node involvement are rare [11], and lymphadenectomy or mesorectal excision is unnecessary. An appropriate segmental en bloc resection is enough for colonic GISTs only if adjacent organs are involved [12]. However, 3 of 17 patients (17.6%) had lymph node metastasis in our present study, which was apparently higher than the previous report. Although the incidence of lymph node metastasis was relatively low, necessity of lymphadenectomy may need careful consideration in the treatment of colonic GISTs.

Even with surgical resection, there is a high risk of recurrence and metastasis. Distant metastases are the most frequent treatment failure for colorectal GISTs and are associated with poor prognosis. The most common site of metastasis in colorectal GISTs is liver, followed by peritoneum. Other locations include pleura, lung, bone and retroperitoneum [13]. In our present study, 9 patients suffered liver metastasis and 7 patients suffered peritoneal metastasis. Whereas GISTs predominantly metastasis to liver and leiomyosarcomas mainly spread to lung [14]. This may assistant in the differential diagnosis of the two tumors.

It was reported that approximately 10-30% of GISTs are regarded as clinically malignant [15], and tumor size and mitotic index are valuable predictors for evaluating malignant potential of GISTs [16]. In the present study, mitotic index more than 5/50HPF and high risk category were associated with poorer prognosis. Tumor location is also a critical risk factor for recurrence after radical surgical resection [17]. However, the modified NIH risk classification system only distinguishes gastric from non-gastric tumors. The prognostic features of colonic GISTs are unclear. Considering different distribution of tumor size and NIH risk category between colonic and gastric GISTs, patients were matched in order to compare the prognosis. The survival analysis showed that the DFS and DSS of colonic GISTs were significantly lower than gastric GISTs. It was reported that non-gastric GISTs have similar risk for tumor recurrence [18]. However, the survival of colonic GISTs were not compared with that of duodenum, small intestine or rectum, due to the limited sample size of GISTs in these locations in our center.

There are some limitations of the present study. First, the present study is a retrospective analysis and lacks systematic prospective data. Therefore, completeness of the data is limited. Second, the sample size of colonic GISTs was not large enough, which will result in sampling error. Third, due to the limited sample size of duodenum, small intestine or rectum GISTs in our center, we could not compare the clinicopathological features and prognosis of colonic with non-gastric GISTs.

**Figure 6:**
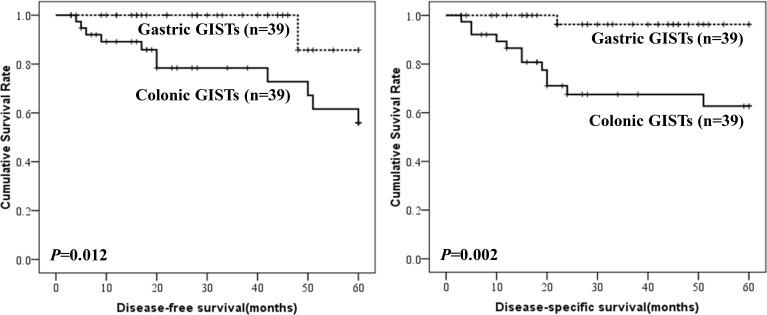
Comparison of DFS and DSS between colonic and gastric GISTs

## CONCLUSIONS

The commonest location for colonic GISTs was sigmoid colon, followed by transverse colon. Most colonic GISTs are high risk category. Mitotic index was the unique independent predictor for the prognosis of colonic GISTs. Colonic GISTs differ significantly from gastric GISTs in respect to clinicopathological features. The prognosis of colonic GISTs was worse than that of gastric GISTs.

## PATIENTS AND METHODS

GISTs cases of the colon were from our department and in addition from the literature. From May 2010 to March 2015, 3 cases of colonic GISTs were diagnosed and received treatment in our department. Literature search of MEDLINNE was performed for all articles in English published from 2000 through 2015. MEDLINNE search resulted in 21 case reports [19–39] including 22 patients and 4 case series [8, 40–42] including 54 cases. To this end, a total of 79 colonic GISTs patients were identified (Figure 1). In addition, the clinicopathological characteristics and prognosis of 297 cases of gastric GISTs were analyzed and compared with colonic GISTs. This study was approved by the Ethics Committee of Xijing Hospital, and written informed consent was obtained from the three patients in our center.

Clinicopathological data including age, gender, accompanied tumor, symptoms, location, tumor size, surgical intervention, histological type, lymph node involvement, mitotic index, immunohistochemical features, mutational status, NIH risk category, adjuvant therapy, tumor recurrence or metastasis and survival data were recorded from hospital medical records or extracted from published reports and studies. The tumors were categorized into very low, low, intermediate and high risk groups according to the modified NIH risk classification criteria [43]. For survival analysis, the exclusion criteria were listed as follows: 1. not receive R0 resection, 2. with tumor rupture during operation, 3. accompanied with distant metastasis, 4. accompanied with GIST in other locations, 5. accompanied with other malignant tumors, 6. with neoadjuvant imatinib therapy, 7. without follow up data. Due to data acquisition, completeness of data is limited.

Data were processed using SPSS 16.0 for Windows (SPSS Inc., Chicago, IL, USA). Numerical variables were expressed as the mean ± SD unless otherwise stated. Discrete variables were analyzed using the Chi-square test or Fisher's exact test. Significant predictors for survival identified by univariate analysis were assessed by multivariate analysis using the logistic regression analysis. Evaluation for disease-free-survival (DFS) and disease-specific-survival (DSS) were obtained by the Kaplan-Meier method. The P value was considered to be statistically significant at the 5% level.
